# Spatiotemporal trends in tetracycline- and trimethoprim–sulfamethoxazole-resistant *S. aureus* among veteran outpatients in the eastern United States

**DOI:** 10.1017/S0950268826101216

**Published:** 2026-02-23

**Authors:** Miah Boyle, Qianyi Shi, Shinya Hasegawa, Margaret Carrel, Jacob Oleson, Michihiko Goto

**Affiliations:** 1 School of Earth, Environment, and Sustainability, University of Iowa, Iowa City, IA, USA; 2 Department of Internal Medicine, University of Iowa, Iowa City, IA, USA; 3 Center for Access & Delivery Research and Evaluation (CADRE), Iowa City Veterans Affairs Health Care System, Iowa City, IA, USA; 4 Department of Biostatistics, University of Iowa, Iowa City, IA, USA

**Keywords:** Staphylococcus aureus, MRSA, antimicrobial resistance, spatiotemporal trends, Bayesian modeling, veteran outpatients

## Abstract

This study examines spatiotemporal patterns of tetracycline- and trimethoprim–sulfamethoxazole (TMP–SMX)–resistant *Staphylococcus aureus* (*S. aureus*) among United States (US) Veterans Health Administration (VHA) outpatients. Prevalence of tetracycline and TMP–SMX resistance in methicillin-susceptible *S. aureus* (MSSA) and methicillin-resistant *S. aureus* (MRSA) was calculated for 2010–2023. MRSA cases from 2018 to 2022 were aggregated to commuting zones (CZs) in the eastern US, and CZ-specific relative risks and temporal trends were estimated using a hierarchical Bayesian Poisson model with a spatiotemporal interaction term. Results indicated that resistance in MRSA increased by 16.4% for tetracycline and 9.3% for TMP–SMX, while MSSA resistance remained stable. High-risk CZs were limited (3% for tetracycline, 4% for TMP–SMX) and distributed across the eastern US, with notable within-state variation in risk and trend. Most CZs exhibited stationary trends, although distinct patterns in the rate and timing of changes in resistance were observed in CZ-specific plots. These evolving and geographically variable patterns of antimicrobial resistance at finer spatial scales highlight the need for local surveillance and outpatient antibiotic stewardship strategies that consider place-based sociodemographic, ecologic, and clinical factors.

## Introduction

Antimicrobial-resistant (AMR) bacterial infections constitute a major public health crisis, causing an estimated 700,000 deaths annually worldwide, with this number projected to rise to 10 million by 2050. [1] Although methicillin-resistant *Staphylococcus aureus* (*S. aureus*) (MRSA) continues to be prevalent in the United States (US), its rates have declined in several patient populations, with a dramatic reduction observed among patients in Veterans Health Administration (VHA) acute care and nursing home facilities. [2–8] Between 2005 and 2017, MRSA rates in VHA inpatients decreased by 55%, driven by the nationwide implementation of the MRSA bundle in 2007. [3, 5] A recent study found similar trends in the US veteran outpatient population, with peak MRSA prevalence of 53.6% in 2010, dropping to 38.8% in 2019. [4]

Despite its overall decline, increased resistance to additional antibiotics in MRSA, such as tetracyclines and trimethoprim–sulfamethoxazole (TMP–SMX), has heightened concerns of multi-drug-resistant strains both domestically and globally. [9–11] In the US veteran outpatient population, tetracycline- and TMP–SMX-resistant community-associated MRSA (CA-MRSA) infections have increased from 2010 to 2019 by 9.2% and 6.6%, respectively. [4] Increases in resistance to non-beta-lactam antibiotics among MRSA have also been seen in the US inpatient and paediatric populations, most notably for clindamycin, mupirocin, and TMP–SMX. [4, 12–16] This is particularly concerning, as many clinicians prescribe non-beta-lactam antibiotics for empirical coverage of MRSA in infections that do not warrant hospitalization.

Current research on *S. aureus* resistance rates to non-beta-lactam antibiotics is limited, largely due to data availability. Nevertheless, geographic variability in temporal trends has been observed across the US for both the prevalence of MRSA and *S. aureus* resistant to non-beta-lactam antibiotics. Regional differences have been reported, with the greatest reductions in MRSA percentage and increases in tetracycline and TMP–SMX resistance found in the south and northeast. [4, 13] Finer-scale analysis revealed that while regional tetracycline and TMP–SMX resistance rates may be rising, these trends often vary among counties within the region. [4] This intra-regional variation suggests that factors beyond patient-level clinical characteristics, such as ecological and socioeconomic differences, shape *S. aureus* resistance patterns, emphasizing the need for analysis at finer spatial scales. Leveraging more spatially representative data, such as VHA electronic health records, can help address this need, providing insights into how geographically variable factors may influence resistance rates.

The aim of this study was to examine the spatiotemporal patterns of the relative risks of tetracycline- and TMP–SMX-resistant MRSA among VHA outpatients in eastern US commuting zones (CZs) from 2018 to 2022. By (1) identifying hotspots of resistant MRSA relative risk and (2) areas where local trends are increasing at a faster rate relative to the overall trend, this study can generate hypotheses about place-based sociodemographic and ecological factors that may be influencing the timing and rate of increase in these areas.

## Methods

### Study population and data source

We obtained data from the VHA’s electronic health record repository, VA Corporate Data Warehouse, for VHA outpatients at 127 medical centres and over 1,400 outreach clinics with positive *S. aureus* cultures from 1 January 2010 to 30 September 2023. The data included patient-level residential information and were linked to microbiology results containing antimicrobial susceptibility data for several antibiotic classes, including tetracyclines and TMP–SMX. Approximately 90% of facilities had on-site microbiology laboratories, required to perform routine quality control and use Food and Drug Administration (FDA)–approved methods in accordance with VHA-designated accreditation organizations. [17, 18]

Inclusion criteria were applied for patients aged 18 years or older, residing in contiguous states of the US or the District of Columbia (DC), with address-level geographic data, and at least one recorded antimicrobial susceptibility result. The data were further filtered to exclude observations with missing MRSA classification and retain only the first observation per 30-day period per patient. The data were stratified into MRSA and methicillin-susceptible *S. aureus* (MSSA) based on resistance, including intermediate resistance, to beta-lactam agents with antistaphylococcal activity, specifically cephalosporins, antistaphylococcal penicillin, or carbapenem. To examine overall trends in resistance, the monthly prevalence of tetracycline and TMP–SMX resistance across the entire US from 2010 to 2023 was calculated and plotted using the ggplot2 version 3.5.1 package in R version 4.4.1.

For the Bayesian analyses, MRSA isolates from 2018 to 2022 were aggregated to annual total counts and counts of cultures resistant to tetracyclines and TMP–SMX at the CZ level to prioritize more recent trends. Focusing on this time period allowed the model to capture recent net changes in relative risk while minimizing the influence of any larger prevalence shifts from earlier in the study period. CZs use commuting data to capture local economics, linking areas to their nearest economic centres. [19] Due to insufficient sample sizes in many western CZs and prior evidence of greater increases in tetracycline and TMP–SMX resistance in the south and northeast, Bayesian analyses were restricted to the eastern US. [4, 13] Of the 593 CZs defined in the 2020 delineation, 331 of the eastern CZs were included in the Bayesian model. [20]

### Statistical analysis

To account for variations across space and time, a hierarchical spatiotemporal Bayesian Poisson model with a log-link function was applied to cases of tetracycline- and TMP–SMX-resistant MRSA within each CZ *i* and year *j:*


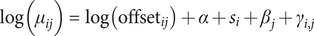



The offset term was the log of the expected count, calculated by multiplying the proportion of tetracycline- or TMP–SMX-resistant cases by the adjusted number of MRSA cases within each CZ and year. The intercept term ( 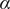 ) represents the mean log risk for the response from 2018 and 2022. The spatially correlated random effect ( 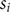 ) follows a Besag–York–Mollié (BYM2) model, including an intrinsic conditional autoregressive (ICAR) component and an unstructured random effect, with the overall variance parameter assigned a weakly informative N(0,1) prior. [21, 22] A temporal effect term ( 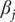 ) estimates the average time trend across all CZs, with year coded as an indexed effect, while the space–time interaction ( 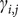 ) estimates any deviation of a CZ’s temporal trend from this average. A weakly informative N(0, 10) prior was assigned to the intercept, temporal effect, and space–time interaction term.

The model was run in Stan version 2.32.2, utilizing the RStan version 2.32.6 R package. [23] Three chains were run for 50,000 iterations, removing the first 25,000 iterations as burn-in and keeping every fifth sample for a total of 15,000 posterior samples. To assess model convergence, the R-hat and number of effective samples ( 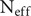 ) were reviewed. The linear predictor ( 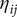 ) was generated, representing the log-relative risk of each CZ and year. Posterior samples for the linear predictor, temporal effect, and space–time interaction terms were extracted, and the mean yearly, CZ, and CZ-year absolute relative risks were calculated. The mean yearly differences in the space–time interaction term were calculated to infer CZ-specific temporal trends.

All CZs were categorized based on their relative risk and temporal trend using a two-step classification procedure. [24] A CZ was classified as high risk if the posterior probability of its relative risk exceeding one was >0.8, moderate risk if between 0.2 and 0.8, and low risk if <0.2. Temporal trends were classified as increasing if the posterior probability of a positive yearly change in the interaction effect was >0.8, stationary if between 0.2 and 0.8, and decreasing if <0.2. An alternative procedure using more lenient cut-offs for temporal trends (>0.6 for increasing; <0.4 for decreasing) was applied to assess whether less stringent criteria improved the sensitivity of CZ classification. The CZ-specific mean relative risks, temporal trends, and classifications were mapped in ArcGIS Pro with the 20 most populated cities based on the 2020 US Census (Esri, Redlands, CA). The yearly absolute relative risks were plotted for the CZs with the three highest posterior probabilities for increasing trend and compared to the average. Model and classification details, additional Stan settings, and R code are provided in the Supplemental Methods.

To evaluate model robustness, we conducted several sensitivity analyses. Potential confounding between the temporal effect term and the space–time interaction term was assessed by comparing the posterior distributions of the temporal effect from the original model and a model fitted without the space–time interaction. The original model specified weakly informative N(0, 10) priors for both the temporal effect and space–time interaction terms. To assess sensitivity to these priors, the model was re-estimated using a non-centred first-order random walk prior (RW1) for the temporal effect and a structured non-centred RW1 prior over time within each CZ for the space–time interaction term. Robustness was evaluated by examining correlations between posterior mean relative risks and the space–time interaction estimates, comparing posterior differences in temporal effects, and reassessing the two-step CZ risk and trend classification relative to the original model. Ethical approval was given by the institutional review board at the University of Iowa and the Research and Development Committee at the Iowa City Veterans Affairs Health Care System with a waiver of informed consent because the study was a retrospective analysis of health records with no direct contact with patients. This study followed the Strengthening the Reporting of Observational Studies in Epidemiology (STROBE) reporting guideline.

## Results

From the 558,737 *S. aureus* culture results compiled from the VHA’s electronic health record repository, 469,624 were included in the analysis. The first result per patient per month was retained (excluding n = 75,842), and patients were excluded if aged less than 18 years (n = 3), resided outside the 48 contiguous states or DC (n = 5,277), were missing geographic data and all antibiotic resistance results (n = 1,517), or lacked MRSA classification (n = 6,474) (Supplementary Table S1).

**Figure 1.**
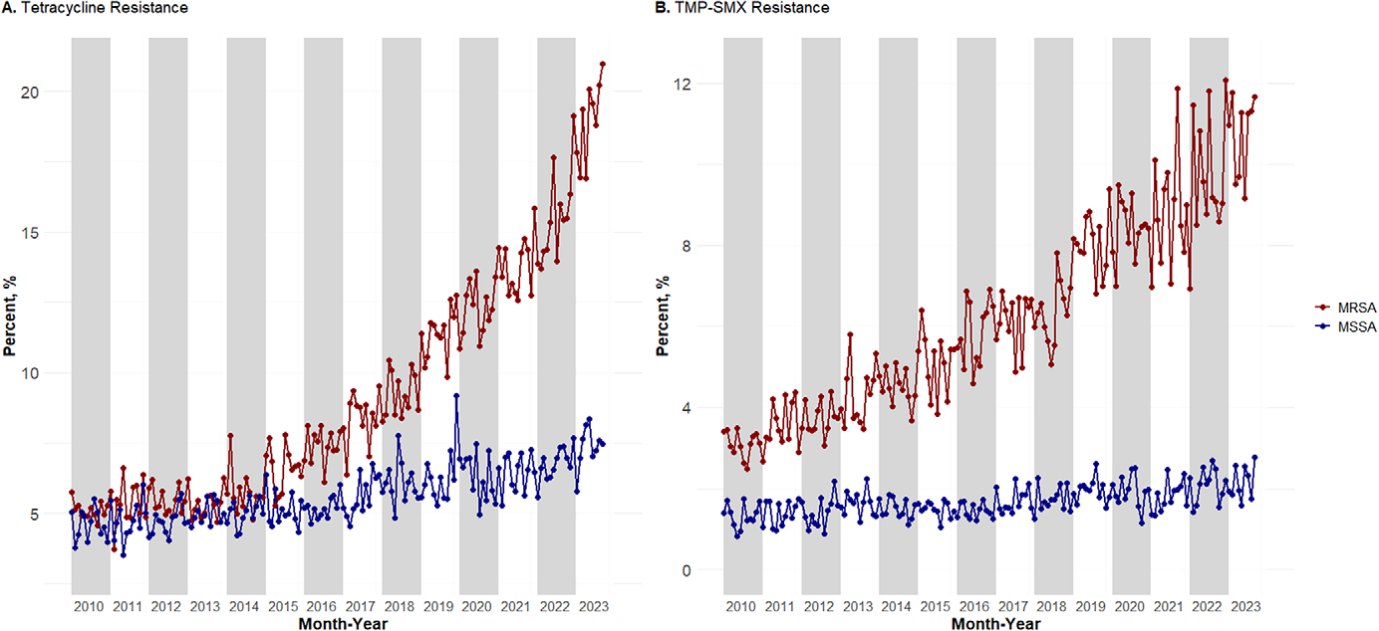
Monthly prevalence from January 2010 to September 2023 for VHA outpatients for (a) tetracycline-resistant MRSA and MSSA and (b) TMP–SMX-resistant MRSA and MSSA.

Prevalence of tetracycline (Figure 1a) and TMP–SMX resistance (Figure 1b) from 2010 to 2023 was plotted using 205,887 MRSA isolates from 149,422 outpatients and 263,737 MSSA isolates from 199,871 outpatients across the entire United States. Tetracycline resistance in MRSA increased from a 2010 low of 4.57% to a 2023 high of 20.98%. TMP–SMX resistance in MRSA increased from a 2010 low of 2.48% to a 2023 high of 11.77%. This trend was not seen in MSSA isolates. Tetracycline resistance in MSSA only varied by approximately 5.66%, with a 2010 low of 3.78% and a 2023 high of 8.35%. TMP–SMX resistance in MSSA had a smaller range of 1.93%, with a 2010 low of 0.83% and a 2023 high of 2.76%.

A hierarchical Bayesian model was fit using aggregated data of 58,901 MRSA isolates collected from 45,325 outpatients between 2018 and 2022. The estimated mean absolute relative risk of tetracycline-resistant MRSA rose from 0.77 (95% confidence interval (95% CI) = 0.58, 1.26) in 2018 to 1.10 (95% CI = 0.85, 1.66) in 2022 (Supplementary Figure S1A). The estimated mean absolute relative risk of TMP–SMX-resistant MRSA also increased from 0.68 (95% CI = 0.51, 1.20) in 2018 to 0.96 (95% CI = 0.73, 1.59) in 2022 (Supplementary Figure S1B).

**Figure 2.**
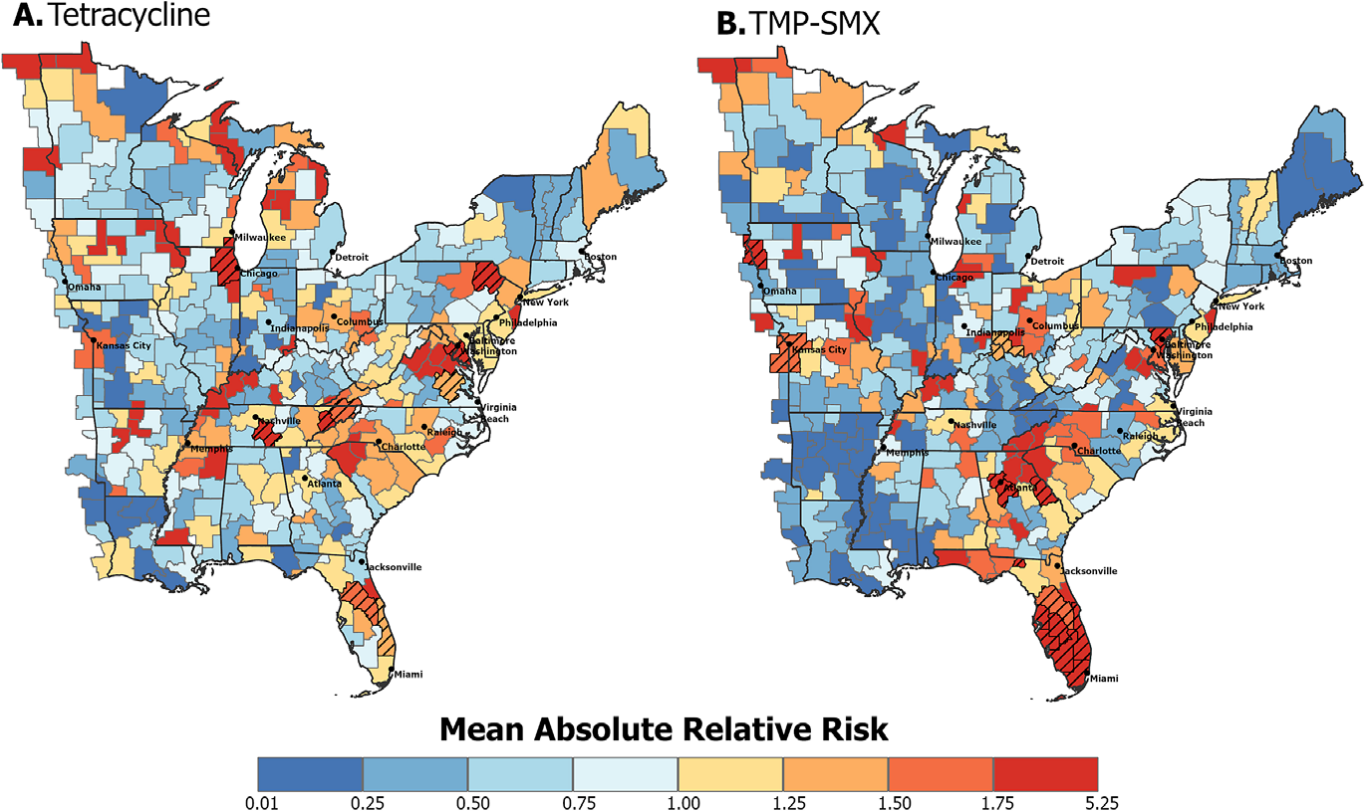
Average absolute relative risk in eastern US commuting zones between 2018 and 2022 of (a) tetracycline resistance and (b) TMP–SMX resistance in MRSA. Hatched commuting zones are classified as high risk based on the first step of the classification procedure described in the methods section, where 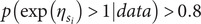 .

The CZ-specific average absolute relative risk for tetracycline (Figure 2a) and TMP–SMX (Figure 2b) resistance in MRSA was geographically heterogeneous. For both resistance types, higher-risk CZs were distributed throughout the study area, in both densely populated metropolitan and rural areas. High-risk hot spots for tetracycline-resistant MRSA were evident along the eastern states and near central metropolitan cities. High-risk CZs for TMP–SMX-resistant MRSA were located near metropolitan cities, with a notable cluster in southern Florida. To quantify the certainty in the mean relative risk estimates, the posterior probabilities of the relative risks being greater than 1 were calculated. High-risk CZs with posterior probabilities greater than 0.8 are visualized with a hatched pattern in Figure 2. For tetracycline-resistant MRSA, CZs categorized as high risk were concentrated along the east coast states, with two centrally located CZs containing or adjacent to metropolitan cities. CZs categorized as high risk for TMP–SMX-resistant MRSA were largely located in South Florida, with additional high-risk CZs containing major metropolitan cities. All CZ-specific posterior probabilities for relative risk being greater than 1 can be visualized in Supplementary Figure S2.

**Figure 3.**
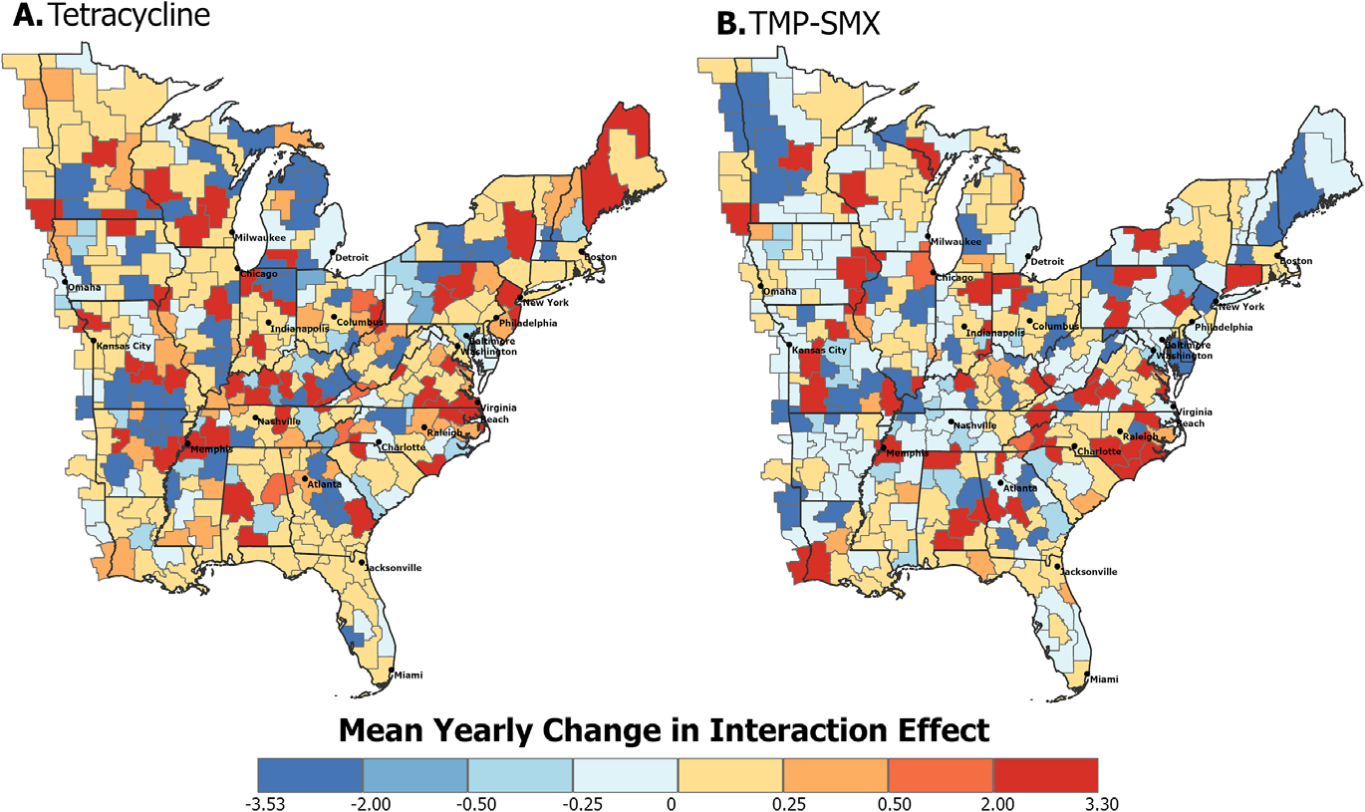
Spatiotemporal trends of eastern US commuting zones between 2018 and 2022. The mean yearly change in the interaction effect for (a) tetracycline resistance and (b) TMP–SMX resistance in MRSA indicates the presence of an overall increasing (positive) or decreasing (negative) trend. The hatched commuting zone in Figure 5a is classified as having an increasing trend based on the second step of the classification procedure described in the methods section, where 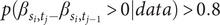 .

The temporal trend of each CZ over the study period is represented by the mean yearly change in the spatiotemporal interaction effect. Positive values suggest increasing trends, while negative values indicate decreasing trends. These spatiotemporal trends show high heterogeneity for both tetracycline-resistant (Figure 3a) and TMP–SMX-resistant MRSA (Figure 3b).

**Figure 4.**
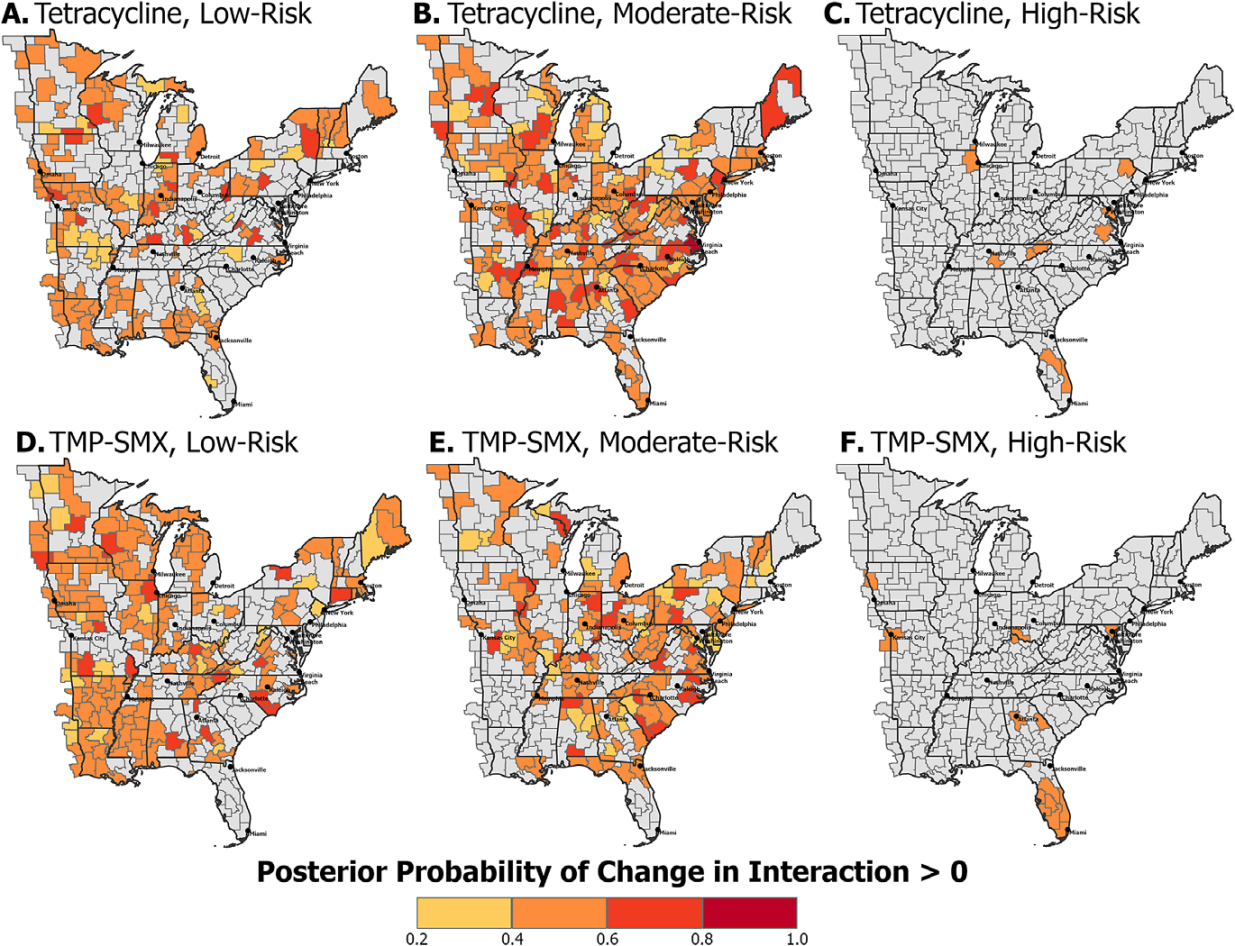
Posterior probabilities of increasing spatiotemporal trends in eastern US commuting zones between 2018 and 2022 stratified by risk group. The darker the colour, the greater the likelihood of that CZ having a positive yearly change in the spatiotemporal interaction term, indicating an overall increasing trend. (a) Low-risk, (b) moderate-risk, and (c) high-risk commuting zones for tetracycline resistance in MRSA. (d) Low-risk, (e) moderate-risk, and (f) high-risk commuting zones for TMP–SMX resistance in MRSA.

To assess the certainty of the spatiotemporal trends, posterior probabilities of the yearly change in the interaction effect being greater than zero were calculated. Visualization of these probabilities was stratified by risk group, determined by the first step of the classification procedure outlined in the methods (Figure 4). Low- and moderate-risk CZs for tetracycline-resistant (Figure 4a,b) and TMP–SMX-resistant (Figure 4d,e) MRSA show posterior probabilities primarily between 0.2 and 0.8. One moderate-risk CZ for tetracycline-resistant MRSA, located along the North Carolina and Virginia border, had a posterior probability of greater than 0.8, indicating a likely increasing trend. CZs classified as high risk for tetracycline-resistant (Figure 4c) and TMP–SMX-resistant (Figure 4f) MRSA had posterior probabilities between 0.4 and 0.6, suggesting stable trends.

CZ risk and trend were categorized based on the classification procedure (Table 1). For tetracycline-resistant MRSA, 9 (3%) were categorized as high risk, 189 (57%) as moderate risk, and 132 (40%) as low risk. Nearly all CZs (99.7%) were categorized as having a stationary trend, with one moderate-risk CZ showing an increasing trend. For TMP–SMX-resistant MRSA, 13 (4%) CZs were categorized as high risk, 140 (42%) as moderate risk, and 178 (54%) as low risk. All CZs were categorized as having a stationary temporal trend. Alternative classifications using more lenient cut-offs for temporal trends (<0.4 and > 0.6) altered the categorization of moderate- and low-risk CZs. However, no high-risk CZs with increasing or decreasing trends were identified, further supporting the finding that high-risk CZs remained stable throughout the study period (Supplementary Table S2).

**Table 1.** Two-step classification of commuting zones based on their absolute relative risk and spatiotemporal trend

	Tetracycline-resistant MRSA				TMP–SMX-resistant MRSA			
	High risk	Moderate risk	Low risk	Total	High risk	Moderate risk	Low risk	Total
Increasing	0	1	0	1	0	0	0	0
Stationery	9	189	132	330	13	140	178	331
Decreasing	0	0	0	0	0	0	0	0
Total	9	190	132	331	13	140	177	330

**Figure 5.**
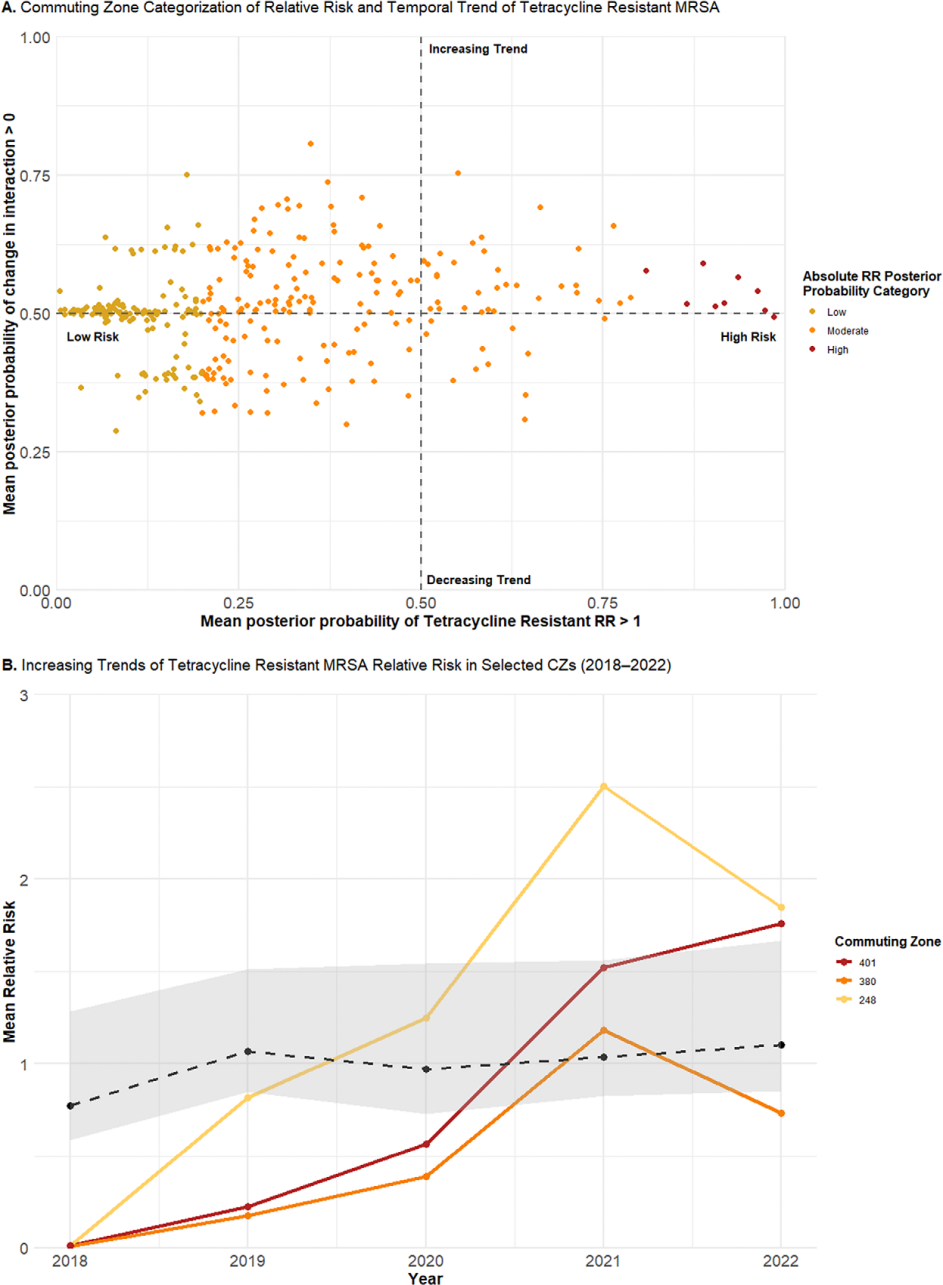
(a) Continuous joint distribution of commuting zone (CZ) mean posterior probabilities of relative risk for tetracycline resistance in MRSA being greater than one (x-axis) and the change in the spatiotemporal interaction term being greater than zero (y-axis). (b) Yearly mean absolute relative risks of tetracycline-resistant MRSA for CZ 401 (Virginia–North Carolina), CZ 380 (New York), and CZ 248 (Maine), plotted alongside the average temporal trend (dotted line) of the study area.

**Figure 6.**
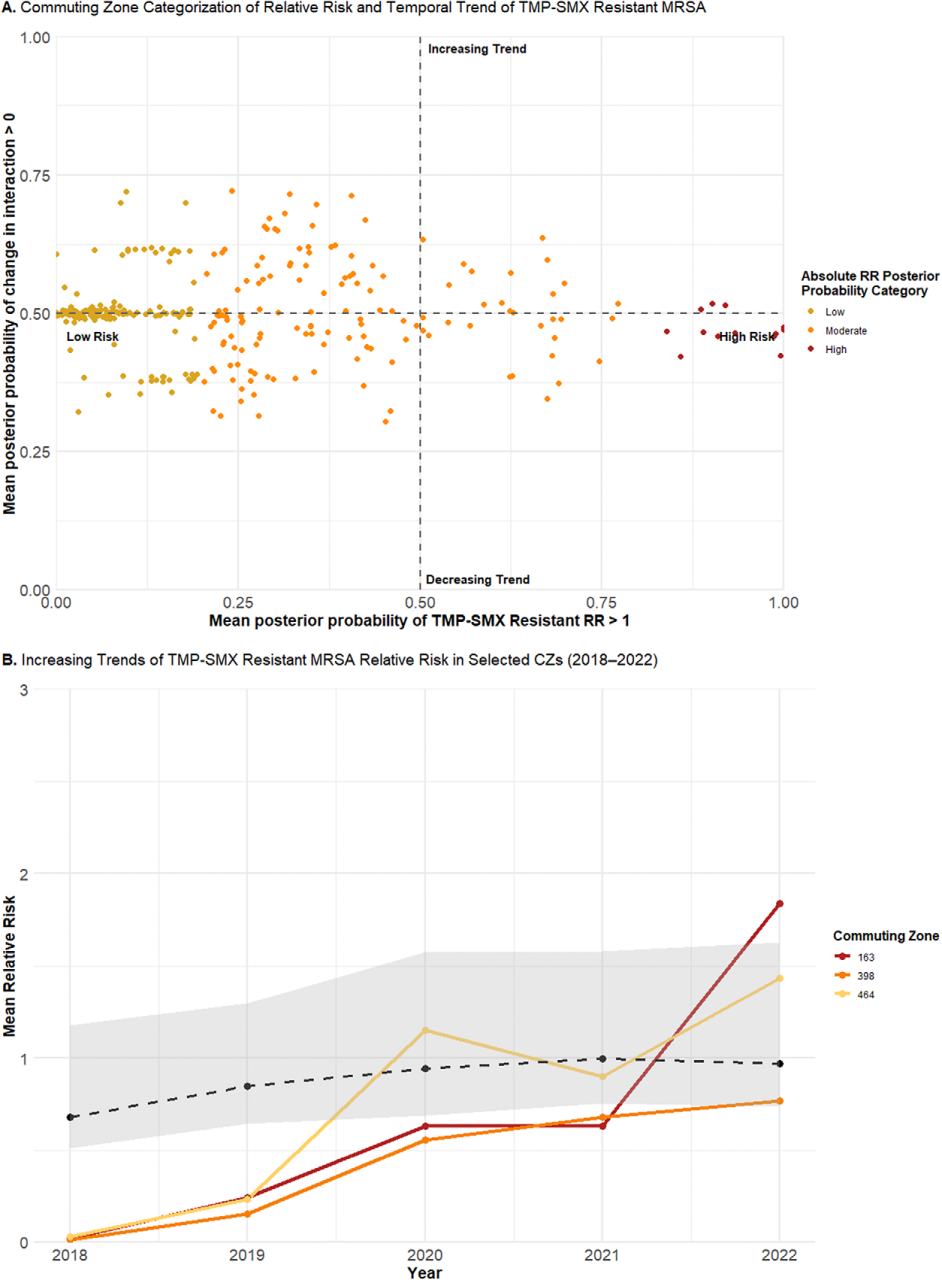
(a) Continuous joint distribution of commuting zone (CZ) mean posterior probabilities of relative risk for TMP–SMX resistance in MRSA being greater than one (x-axis) and the change in the spatiotemporal interaction term being greater than zero (y-axis). (b) Yearly mean absolute relative risks of TMP–SMX-resistant MRSA for CZ 163 (Indiana), CZ 398 (North Carolina), and CZ 464 (Pennsylvania), plotted alongside the average temporal trend (dotted line) of the study area.

Although categorization facilitates straightforward interpretation, aggregating to discrete categories obscures finer differences, particularly in temporal trends. To capture this variation, the continuous distribution of the posterior probabilities was plotted for tetracycline-resistant (Figure 5a) and TMP–SMX-resistant (Figure 6a) MRSA. Low-risk CZs for both resistance types were concentrated around a posterior probability of a positive change in the spatiotemporal interaction term of 0.5, consistent with a stationary trend. In contrast, moderate-risk CZs, as well as low-risk CZs near the 0.2 threshold, demonstrated greater variability in their posterior probabilities of increasing trends. High-risk CZs also remained near a posterior probability of 0.5, suggesting a likely stationary pattern.

The yearly mean absolute relative risks for the three CZs with the highest posterior probability of increasing trends were plotted. For tetracycline-resistant MRSA, these included CZ 401 (Virginia–North Carolina), CZ 380 (New York), and CZ 248 (Maine) (Figure 5b). All showed increasing trends from 2018 to 2022, with a sharp increase between 2020 and 2021. CZs 401 and 248, categorized as moderate risk, showed trends rising above the average, whereas CZ 380 remained mostly below the average, consistent with its categorization of low risk despite an increasing trend.

For TMP–SMX-resistant MRSA, the three CZs with the highest posterior probabilities of increasing trends included CZ 163 (Indiana), CZ 398 (North Carolina), and CZ 464 (Pennsylvania) (Figure 6b). All exhibited increasing trends from 2018 to 2022, though the rate and timing of change varied. CZ 163 exhibited a steady increase, remaining below the average trend until an almost fivefold increase between 2021 and 2022. CZ 398 increased at a gradual rate, with a steep increase between 2019 and 2020, though it remained below the average trend, consistent with its low-risk categorization. In contrast, CZ 464 showed a fluctuating trend, with sharp increases elevating it above the average trend in 2020 and 2022, and a decrease between 2020 and 2021, temporarily bringing it below the average.

Sensitivity analyses indicated that the primary findings were robust to alternative model specifications. Posterior distributions of the main temporal effect were nearly identical between models fitted with and without the space–time interaction, with posterior mean differences centred near zero and 95% CIs overlapping zero across all years, indicating minimal confounding between terms (Supplementary Table S3). Re-estimation of the models using non-centred RW1 priors resulted in CZ-level posterior mean relative risks that were highly correlated with those from the original model (Spearman’s ρ = 0.87–0.92; Pearson’s r = 0.85–0.88), with slightly lower but positive correlations for the space–time interaction term (Spearman’s ρ = 0.74–0.81; Pearson’s r = 0.73–0.78). Posterior differences in the temporal effect under RW1 prior were small and again included zero in the 95% CIs across all years (Supplementary Table S4). Although reclassification resulted in more CZs categorized as high and low risk, none were reclassified as having an increasing or decreasing temporal trend, and the overall pattern of temporal stability remained unchanged (Supplementary Table S5).

## Discussion

Electronic health record data from a nationwide sample of VHA outpatients demonstrated that the prevalence of tetracycline- and TMP–SMX-resistant MRSA increased by 16.41% and 9.29%, respectively, from 2010 to 2023, consistent with previous studies. [4, 13, 15] This rise was not mirrored in MSSA, suggesting fundamental differences in their genetic compositions and epidemiology. These patterns, along with the emergence of new CA-MRSA strains and the spread of healthcare-associated (HA-MRSA) strains into the general population, suggest the presence of a large reservoir of MRSA outside healthcare facilities, with antimicrobial resistance patterns being shaped by a multitude of selective pressures. [25] At the same time, the long-dominant CA-MRSA clone USA300 has declined in certain regions since its peak in the mid-to-late 2000s, potentially facilitating clonal replacement by more resistant strains during the mid-2010s. [26] Changes in prescribing practices may have further amplified these trends, as tetracycline use has increased in recent years among US outpatients. [27] These findings highlight the need for antibiotic stewardship strategies that address evolving resistance patterns, particularly for antibiotics commonly used in outpatient settings, as well as infection control strategies implemented in both community and healthcare settings.

The geographic distributions of relative risk and temporal trend were highly heterogeneous, with variation observed within states. This suggests that the risk of resistant MRSA infections is more likely driven by local, rather than broader statewide factors, emphasizing the importance of examining underlying place-based factors. While the use of antibiotics remains the primary driver of resistance, sociodemographic factors such as household crowding and poor health literacy have been shown to contribute to racial and economic health disparities in AMR bacterial infections across the US. [28–32] These disparities have been linked to neighbourhood characteristics, such as education and rurality, with significant clustering of resistant infections observed in areas of high deprivation. [33, 34] Climate factors can further influence patient behaviour and their interactions with their environment, likely contributing to the higher rates of resistance observed in the south. [35, 36] The identification of CZs with elevated risk raises questions about which local-level sociodemographic, environmental, or public health factors may be driving these differences, underscoring the importance of local-level surveillance to inform future studies.

Although high- and low-risk CZs exhibited relatively stable temporal trends, CZ-specific relative risk plots revealed distinct temporal patterns in the rate and timing of change, highlighting the importance of localized surveillance, as larger spatial scales may obscure these differences. Several notable changes in relative risk were observed, particularly during the post-COVID-19 period from 2020 to 2022. In all three CZs examined for trends in tetracycline-resistant MRSA, increases were observed between 2020 and 2021. The timing and magnitude of these increases may reflect local factors or COVID-19-related disruptions, including shifts in prescribing practices and changes in healthcare delivery. While several studies reported decreased antibiotic prescriptions during the pandemic’s first wave, the sharp decline in in-person visits, except in patients with more severe infections, may have contributed to the underdiagnosis of bacterial infections, inflated resistance rates, and suboptimal antibiotic use. [37–41]

This study has several limitations. Although the VHA provides a nationwide sample with standardized microbiology testing, it is not representative of the broader US population, as VHA patients are more likely to be male gender, older in age, and White race. Aggregating to finer geographic units, such as CZs, results in low or zero counts, particularly in the Mountain West Region, requiring the analysis to be restricted to the eastern US. Additionally, use of a classification procedure for categorizing temporal trends, while conceptually advantageous, is better suited to detecting linear trends. High posterior probabilities are most likely in CZs with consistent increases over time, reducing the method’s sensitivity to fluctuating or non-linear trends. As demonstrated in the results, CZ-specific trends often exhibit non-linearity, which is better captured by examining posterior probabilities on a continuous scale rather than through discrete categorization.

Despite the decline of MRSA in the US, it remains a prominent cause of infection, with growing concerns over rising resistance to additional, commonly utilized antibiotics. This study characterizes the spatiotemporal patterns of relative risk for tetracycline- and TMP–SMX-resistant MRSA in the eastern US, identifying CZs with elevated risk and increasing local trends. In doing so, the findings illustrate how finer spatial scales can capture local variation that may be obscured at more broadly aggregated geographic units. The identification of stable yet spatially heterogenous patterns in tetracycline- and TMP–SMX-resistant MRSA points towards the influence of local, place-based factors, rather than broader statewide determinants. This highlights the need for a deeper understanding of how sociodemographic, ecological, and healthcare characteristics, particularly in the wake of the COVID-19 pandemic, shape the geographic distribution and temporal trends of AMR.

## Supporting information

10.1017/S0950268826101216.sm001 Boyle et al. supplementary material Boyle et al. supplementary material

## Data Availability

The data that support the findings of this study are from the US VHA electronic health record repository. Restrictions apply to the availability of the data, which contain protected health information and are not publicly available. Access may be granted with appropriate VHA approval. The R code used in this study is available in the Supplementary Methods.

## Supplementary material

The supplementary material for this article can be found at http://doi.org/10.1017/S0950268826101216.

## References

[1] World Health Organization B (2019) No Time to Wait: Securing the Future from Drug-Resistant Infections. Geneva, Switzerland: World Health Organization.

[2] Evans ME, et al. (2017) Eight years of decreased methicillin-resistant Staphylococcus aureus health care-associated infections associated with a veterans affairs prevention initiative. American journal of infection control. 45(1), 13–16. 28065327 10.1016/j.ajic.2016.08.010

[3] Jain R, et al. (2011) Veterans affairs initiative to prevent methicillin-resistant Staphylococcus aureus infections. New England Journal of Medicine. 364(15), 1419–1430. 21488764 10.1056/NEJMoa1007474

[4] Carrel M, et al. Antimicrobial resistance patterns of outpatient Staphylococcus aureus isolates. JAMA Network Open. 2024;7(6):e2417199-e. 38874923 10.1001/jamanetworkopen.2024.17199 PMC11179135

[5] Jones M (2019) Vital signs: Trends in *Staphylococcus aureus* infections in veterans affairs medical centers—United States, 2005–2017. MMWR Morbidity and Mortality Weekly Report. 68(9), 220–224. 30845116 10.15585/mmwr.mm6809e2 PMC6421970

[6] Klevens RM, et al. (2007) Invasive methicillin-resistant Staphylococcus aureus infections in the United States. Journal of the American Medical Association 298(15), 1763–1771. 17940231 10.1001/jama.298.15.1763

[7] Klein E, Smith DL and Laxminarayan R (2009) Community-associated methicillin-resistant Staphylococcus aureus in outpatients, United States, 1999–2006. Emerging Infectious Diseases. 15(12), 1925. 19961671 10.3201/eid1512.081341 PMC3044510

[8] Hidron AI, et al. (2008) Antimicrobial-resistant pathogens associated with healthcare-associated infections: Annual summary of data reported to the National Healthcare Safety Network at the Centers for Disease Control and Prevention, 2006–2007. Infection Control & Hospital Epidemiology. 29(11), 996–1011. 18947320 10.1086/591861

[9] Lynch JP and Zhanel GG (2023) Escalation of antimicrobial resistance among MRSA part 1: Focus on global spread. Expert Review of Anti-infective Therapy. 21(2), 99–113. 36470275 10.1080/14787210.2023.2154653

[10] Guo Y, et al. (2020) Prevalence and therapies of antibiotic-resistance in Staphylococcus aureus. Frontiers in Cellular and Infection Microbiology. 10, 107. 32257966 10.3389/fcimb.2020.00107 PMC7089872

[11] Diekema DJ, et al. (2001) Survey of infections due to staphylococcus species: Frequency of occurrence and antimicrobial susceptibility of isolates collected in the United States. Canada, Latin America, Europe, and the Western Pacific Region for the SENTRY Antimicrobial Surveillance Program, 1997–1999. Clinical Infectious Diseases. 32(Supplement_2), S114–S132. 11320452 10.1086/320184

[12] Sutter DE, et al. (2016) Changing susceptibility of Staphylococcus aureus in a US pediatric population. Pediatrics. 137(4), e20153099. 26933211 10.1542/peds.2015-3099

[13] Ham DC, et al. (2023) Trimethoprim-sulfamethoxazole resistance patterns among Staphylococcus aureus in the United States, 2012–2018. Infection Control & Hospital Epidemiology. 44(5), 794–797. 35166197 10.1017/ice.2022.9 PMC10150455

[14] Tickler IA, et al. (2017) Continued expansion of USA300-like methicillin-resistant Staphylococcus aureus (MRSA) among hospitalized patients in the United States. Diagnostic Microbiology and Infectious Disease. 88(4), 342–347. 28529090 10.1016/j.diagmicrobio.2017.04.016

[15] Khamash DF, et al. (2019) Increasing clindamycin and trimethoprim-sulfamethoxazole resistance in pediatric Staphylococcus aureus infections. Journal of the Pediatric Infectious Diseases Society. 8(4), 351–353. 30011009 10.1093/jpids/piy062

[16] Sader HS, et al. (2016) Antimicrobial susceptibility patterns of community-and hospital-acquired methicillin-resistant Staphylococcus aureus from United States hospitals: Results from the AWARE Ceftaroline surveillance program (2012–2014). Diagnostic Microbiology and Infectious Disease. 86(1), 76–79. 27394637 10.1016/j.diagmicrobio.2016.06.017

[17] US Department of Veterans Affairs, Veterans Health Administration (2008) VHA Handbook 1106.01, Pathology and Laboratory Medicine Service Procedures. Washington, DC.

[18] US Food and Drug Administration (2025) Antibacterial Susceptibility Test Interpretive Criteria. https://www.fda.gov/drugs/development-resources/antibacterial-susceptibility-test-interpretive-criteria.

[19] Tolbert CM, Killian MS (1987) Labor Market Areas for the United States. Washington, DC: US Department of Agriculture, Economic Research Service.

[20] Fowler CS (2024) New commuting zone delineation for the US based on 2020 data. Scientific Data. 11(1), 975. 39242662 10.1038/s41597-024-03829-5 PMC11379817

[21] Besag J, York J and Mollié A (1991) Bayesian image restoration, with two applications in spatial statistics. Annals of the Institute of Statistical Mathematics. 43(1), 1–20.

[22] Simpson D, et al. (2017) Penalising Model Component Complexity: A Principled, Practical Approach to Constructing Priors. Statistical Science. 32(1), 1–28.

[23] Stan Development T (2018) RStan: The R interface to Stan. R package version 217, 3.

[24] Li G, et al. (2014) Space–time variability in burglary risk: A Bayesian spatio-temporal modelling approach. Spatial Statistics. 9, 180–191.

[25] David MZ and Daum RS (2010) Community-associated methicillin-resistant Staphylococcus aureus: Epidemiology and clinical consequences of an emerging epidemic. Clinical Microbiology Reviews. 23(3), 616–687. 20610826 10.1128/CMR.00081-09 PMC2901661

[26] Planet PJ (2017) Life after USA300: The rise and fall of a superbug. The Journal of Infectious Diseases. 215(suppl_1), S71–S77. 28375517 10.1093/infdis/jiw444 PMC5853207

[27] Kim C, et al. (2025) Public health surveillance of outpatient antibiotic prescription trends, United States, 2011-2019. American Journal of Epidemiology. 194(10), 3062–3065. 39367708 10.1093/aje/kwae391 PMC12120937

[28] Endale H, Mathewos M and Abdeta D (2023) Potential causes of spread of antimicrobial resistance and preventive measures in one health perspective-a review. Infection and Drug Resistance. 16, 7515–7545. 38089962 10.2147/IDR.S428837 PMC10715026

[29] Nadimpalli ML, Chan CW and Doron S (2021) Antibiotic resistance: A call to action to prevent the next epidemic of inequality. Nature Medicine. 27(2), 187–188. 10.1038/s41591-020-01201-9 PMC7961885 33462445

[30] Olesen SW and Grad YH (2018) Racial/ethnic disparities in antimicrobial drug use, United States, 2014–2015. Emerging Infectious Diseases. 24(11), 2126. 30334733 10.3201/eid2411.180762 PMC6199984

[31] Casale E, et al. (2022) Association between health inequalities, antibiotic use and antibiotic-resistant infections in high-income countries: A scoping review. International Journal of Pharmacy Practice. 30(Supplement_2), ii10–ii11.

[32] Immergluck LC, et al. (2019) Geographic surveillance of community associated MRSA infections in children using electronic health record data. BMC Infectious Diseases. 19, 1–12. 30777016 10.1186/s12879-019-3682-3 PMC6378744

[33] See I, et al. Socioeconomic factors explain racial disparities in invasive community-associated methicillin-resistant Staphylococcus aureus disease rates. Clinical Infectious Diseases. 2017;64(5):597–604. 28362911 10.1093/cid/ciw808 PMC5656382

[34] Cooper LN, et al. (2024) Socioeconomic disparities and the prevalence of antimicrobial resistance. Clinical Infectious Diseases. 79(6), 1346–1353. 38845562 10.1093/cid/ciae313 PMC11650857

[35] Sun L, Klein EY and Laxminarayan R (2012) Seasonality and temporal correlation between community antibiotic use and resistance in the United States. Clinical Infectious Diseases. 55(5), 687–694. 22752512 10.1093/cid/cis509

[36] Klein EY, et al. (2013) The changing epidemiology of methicillin-resistant Staphylococcus aureus in the United States: A national observational study. American Journal of Epidemiology. 177(7), 666–674. 23449778 10.1093/aje/kws273

[37] King LM, et al. (2021) Trends in US outpatient antibiotic prescriptions during the coronavirus disease 2019 pandemic. Clinical Infectious Diseases. 73(3), e652–e660. 33373435 10.1093/cid/ciaa1896 PMC7799289

[38] Buehrle DJ, et al. (2020) Impact of the coronavirus disease 2019 pandemic on outpatient antibiotic prescriptions in the United States. Open Forum Infectious Diseases. 7(12), ofaa575. 33409334 10.1093/ofid/ofaa575 PMC7765437

[39] Hamilton A, et al. (2023) COVID-19 and outpatient antibiotic prescriptions in the United States: A county-level analysis. Open Forum Infectious Diseases. 10(3), ofad096. 36949878 10.1093/ofid/ofad096 PMC10026546

[40] Baum A, Kaboli PJ and Schwartz MD (2021) Reduced in-person and increased telehealth outpatient visits during the COVID-19 pandemic. Annals of Internal Medicine. 174(1), 129–131. 32776780 10.7326/M20-3026 PMC7429994

[41] Baker MA, et al. (2022) The impact of coronavirus disease 2019 (COVID-19) on healthcare-associated infections. Clinical Infectious Diseases. 74(10), 1748–1754. 34370014 10.1093/cid/ciab688 PMC8385925

