# Supplementary material for: Spatiotemporal trends in tetracycline- and trimethoprim–sulfamethoxazole-resistant S. aureus among veteran outpatients in the eastern United States
Source: Epidemiol Infect. 2026 Feb 23;154:e31. doi: 10.1017/S0950268826101216 (PMC12976086; doi:10.1017/S0950268826101216)

Supplementary Figure S1. A) Average temporal trend of the absolute relative risk of tetracycline resistance in MRSA from 2018-2022 within the study area. B) Average temporal trend of the absolute relative risk of TMP-SMX resistance in MRSA from 2018-2022 within the study area.


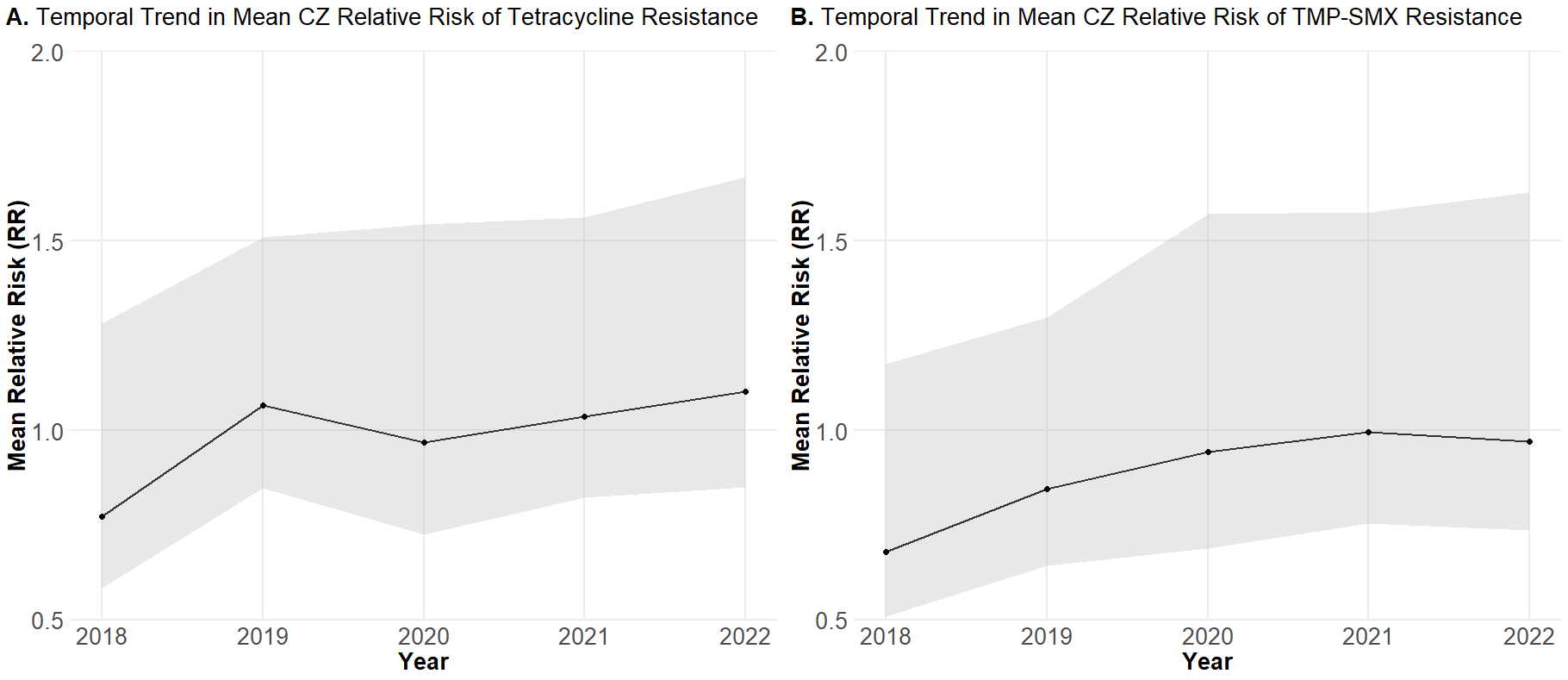


Supplementary Figure S2. Posterior probability that the average absolute relative risk in eastern US commuting zones between 2018 and 2022 is greater than one for A) tetracycline resistance and B) TMP-SMX resistance in MRSA. Commuting zones in the darkest red color have a posterior probability of greater than 0.8, indicating a higher likelihood of increased risk.


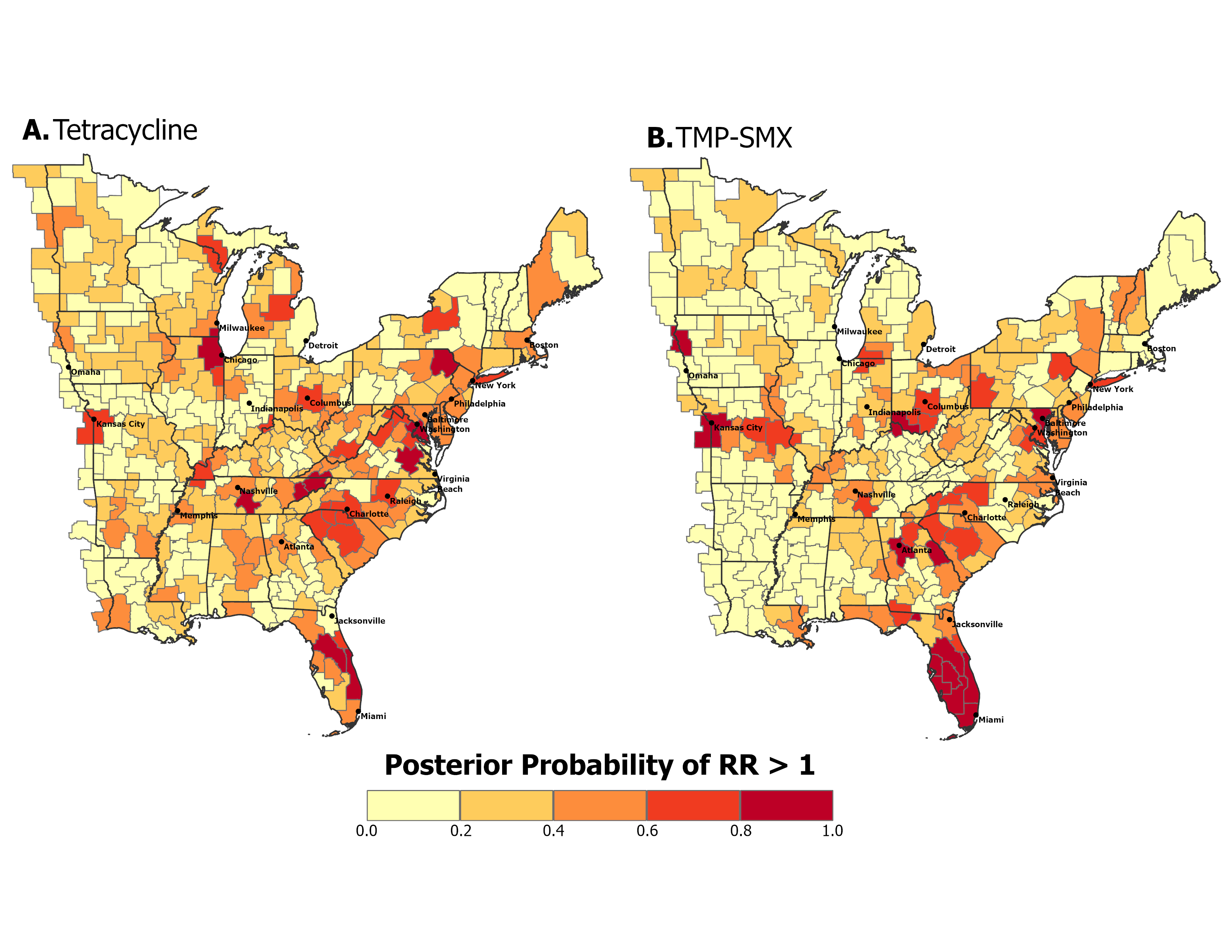

Supplement: Boyle et al. supplementary material [file S0950268826101216sup001.zip › Supplementary Figure S1.docx]
