# Supplementary material for: Spatiotemporal trends in tetracycline- and trimethoprim–sulfamethoxazole-resistant S. aureus among veteran outpatients in the eastern United States
Source: Epidemiol Infect. 2026 Feb 23;154:e31. doi: 10.1017/S0950268826101216 (PMC12976086; doi:10.1017/S0950268826101216)
Supplement: Boyle et al. supplementary material [file S0950268826101216sup001.zip › E&I_final_supplemental_methods.docx]

**Supplemental Methods**

*Commuting Zones*

Commuting zones (CZs), first established in 1987 by the US Department of Agriculture’s Economic Research Service (USDA ERS), are geographic units designed to capture local economics, especially amongst rural areas, linking them to their nearest economic centers.[1] This study uses the 2020 CZ delineations produced by Fowler [2], which were constructed using data on journey to work counts, core-based statistical areas (CBSA), and county boundaries accessed from the Census. The delineation process involved calculating proportional flows, or the connection between counties based on commuter proportions, and using hierarchical clustering to define a total of 593 CZs.[3, 4] All data and documentation for the 2020 CZs are available at <https://github.com/csfowler/CommutingZones2020.git>. The analysis was limited to 336 eastern CZs. Of those 336 CZs, 331 CZs had non-zero counts of *S. aureus* cultures for at least one of the five years included in the study. The CZs excluded due to zero counts of S. aureus cultures for all five years were CZs 541, 291, 285, 259, and 257.

*Spatial Model*

A hierarchical spatiotemporal Bayesian Poisson regression model was applied, with *i* indexing each spatial unit (CZ) and *j* indexing each temporal unit (year). By incorporating a temporal trend common to all spatial units and a space-time interaction term capturing local deviations from the average, this approach enables the detection of disease risk hotspots, examines their evolution over time, and identifies areas with increasing local trends that may indicate emerging hotspots.[5, 6] The full model specification and parameter definitions are described in the main manuscript.

A spatially correlated random effect ($s_{i})$ term is included in the model and follows a BYM2 model. The BYM2 model is a reparameterization of the Besag, York and Mollié (BYM) model, which includes both an intrinsic conditional autoregressive (ICAR) component ($\phi_{i}$) to achieve spatial smoothing and a random effect component ($\theta_{i}$) that accounts for any non-spatially structured heterogeneity between units.[7, 8] In the BYM model, the spatially structured component cannot be assessed independently from the spatially unstructured one, introducing challenges in defining hyperparameters for the two components.[9] The BYM2 model addresses the issue of parameterization in the BYM model by including a single precision parameter and a mixing parameter to model the amount of variance from the two components.[9] The combined random effects for the BYM2 model can be written as:

$$s_{i}= \phi_{i}+ \theta_{i}$$

$$\phi_{i}+ \theta_{i}=\left( \sqrt{p}{\phi_{i}}^{'}+ \left( \sqrt{1-p} \right){\theta_{i}}^{'} \right)\sigma$$

Where $p\in[0, 1]$ is the mixing parameter that models the amount of variance from the spatial correlated error terms and the spatially independent error terms with a prior distribution of beta(0.5, 0.5). The overall standard deviation ($\sigma)$ for the combined terms and the spatially unstructured random effect term (${\theta_{i}}^{'}),$ have a normal prior distribution of N(0,1). The spatially structured random effect term, ${\phi_{i}}^{'}$, captures the spatial dependency across areal units through an ICAR prior. The joint prior of the ICAR model computes the squared differences between neighbors *i* and *j*, penalizing larger differences and resulting in increased spatial smoothing.[10]

$$p\left( \phi\right)\propto\exp\left( -\frac{1}{2}\sum_{i\sim j} ({\phi_{i}-\phi_{j})}^{2} \right)$$

Spatial neighbors were defined for each CZ by queens’ contiguity, requiring neighbors to share a geographic edge or corner. This was used to construct an adjacency matrix using the spdep version 1.3.6 R package.[11] For inclusion in the Bayesian model, the adjacency matrix was converted into two lists, where each pair of entries represents a unique neighbor pairing.

*Bayesian Model Simulation using Stan*

The model was run in Stan version 2.32.2, utilizing the RStan version 2.32.6 R package.[12] Stan implements Hamiltonian Monte Carlo (HMC) No U-Turn (NUTS) sampling to draw from the posterior distribution.[10] The superior efficiency of HMC samplers compared to Gibbs and Metropolis samples allow for better estimates of posterior distributions from more complex models, such as those containing spatial models and spatiotemporal interaction components.[13] A weakly-informative normal prior distribution of N(0, 10) was assigned to the intercept, temporal effect term, and the space-time interaction term. Three chains were run for 50,000 iterations, with the first 25,000 iterations removed as burn-in, keeping every fifth sample for a total of 15,000 posterior samples. The chains were run in parallel with a maximum tree depth of 15 to allow for increased sampler exploration, and parameters were initialized as 0.1 to enforce positive estimates. To assess model convergence and estimate precision, the R-hat and number of effective samples ($N_{\mathrm{eff}})$ were reviewed. The R-hat statistic assesses convergence of the model.[10] All R-hat values were close to one and no greater than 1.1, indicating chain convergence. As the $N_{\mathrm{eff}}$ value increases the Monte Carlo sample error approaches zero, meaning that the estimated parameter mean is closer to the true mean.[10] All values of $N_{\mathrm{eff}}$ were large and no lower than 400, indicating adequate precision of parameter estimates.

*Classification Procedure*

All CZs were categorized based on their relative risk and temporal trend using a two-step procedure, following the approach described in Li, Haining [5]. Classification rules based on posterior probabilities that the relative risk exceeds one, using a 70-80% cutoff, have been shown to yield reasonable sensitivity for moderate expected counts and excess risk.[14] The linear predictor ($\eta_{ij})$ was generated from the model, representing the log-relative risk of each CZ *i* and year *j*, and is defined as the sum of the intercept ($\alpha)$, the spatial random effect ${(s}_{i})$, the fixed effect for year ($\beta_{j})$, and the space-time interaction term ($\gamma_{i, j})$.

$$\eta_{ij}= \alpha+ s_{i}+\beta_{j}+ \gamma_{i, j}$$

First, to classify based on risk, the mean linear predictor across all years for each CZ *i* ($\bar{\eta}_{i}$), representing its average log-relative risk over the study period, was used. A CZ was classified as high risk if the posterior probability $p\left( \exp(\bar{\eta}_{i})>1 | data \right)>0.8$, moderate risk if the posterior probability $0.2<p\left( \exp(\bar{\eta}_{i})>1 | data \right)<0.8$, and low risk if the posterior probability $p\left( \exp(\bar{\eta}_{i})>1 | data \right)<0.2$. Secondly, a CZ was classified as having an increasing trend if the posterior probability $p\left( \gamma_{i, j}{- \gamma}_{i, j-1}>0 \right|data)>0.8$, a stationary trend if the posterior probability $0.2<p\left( \gamma_{i, j}- \gamma_{i, j-1}>0 \right|data)<0.8$, and a decreasing trend if the posterior probability $p\left( \gamma_{i, j}- \gamma_{i, j-1}>0 \right|data)<0.2$. An alternative procedure with more lenient cutoffs of greater than 0.6 for increasing trends, and less than 0.4 for decreasing trends, was also applied to assess whether the less stringent criteria improved the sensitivity of CZ classification in these categories.

*R Code*

## CONSTRUCT CZ ADJACENCY MATRIX

#load cz shapefile:

cz_shape<- st_read("east_cz20.shp")

#Filter to only keep CZ20s that are in east_cz_data

filtered_cz_shape<- cz_shape %>%

filter(CZ20 %in% east_cz_data$CZ20)

# Arrange the dataset by cz id code

filtered_cz_shape <- filtered_cz_shape %>% arrange(CZ20)

#extract cz ids for future posterior prob datasets

cz_id<- filtered_cz_shape$CZ20

#Create spatial neighbors using queen contiguity

cz_nb<- poly2nb(filtered_cz_shape, row.names = cz_id)

#Create an adjacency matrix

adj_matrix<- nb2mat(cz_nb, style = "B")

#Convert from adjacency matrix to node1 and node2, representing neighboring czs

mungeCARdata4stan<- function(adj_matrix) {

node_list<- which(adj_matrix == 1, arr.ind = TRUE)

edges<- as.data.frame(node_list)

colnames(edges)<- c("node1", "node2")

edges<- edges[edges$node1 < edges$node2, ] #keeps unique edges only

list(

N = nrow(adj_matrix),

node1 = edges$node1,

node2 = edges$node2,

N_edges = nrow(edges)

)

}

nbs<- mungeCARdata4stan(adj_matrix)

## PREPARE DATA FOR STAN

#ensure correct indexing of czs: matches between nodes and cz ids:

cz_id_order<- data.frame(

cz_id<- as.integer(filtered_cz_shape$CZ20),

cz_index= 1:length(filtered_cz_shape$CZ20)

)

colnames(cz_id_order)[1]<- "CZ20"

east_cz_data<- east_cz_data %>%

left_join(cz_id_order, by= "CZ20")

#Calculate the expected count for offset term

total_tetR_cases<- sum(east_cz_data$cz_tetra_count) #total cases in the study

total_mrsa_cases<- sum(east_cz_data$cz_mrsa_count) #total population of interest

overall_proportion<- total_tetR_cases/total_mrsa_cases

#to avoid logging zero values:

constant_to_add <- 0.1 # Adjust based on your data scale

east_cz_data$adjusted_mrsa_count <- ifelse(east_cz_data$cz_mrsa_count == 0, constant_to_add, east_cz_data$cz_mrsa_count)

east_cz_data$expected_count<- east_cz_data$adjusted_mrsa_count * overall_proportion

# Calculating the scaling factor for BYM2 ICAR

# Construct the sparse adjacency matrix from node1 and node2 lists

adj_matrix_sparse <- sparseMatrix(

i = nbs$node1,

j = nbs$node2,

x = 1,

dims = c(nbs$N, nbs$N),

symmetric = TRUE

)

# Compute the ICAR precision matrix (Q = D - W)

D <- Diagonal(nbs$N, rowSums(adj_matrix_sparse)) # Diagonal matrix of degrees

Q <- D - adj_matrix_sparse # ICAR precision matrix

# Add a small jitter to the diagonal for numerical stability

Q_pert <- Q + Diagonal(nbs$N) * max(diag(Q)) * sqrt(.Machine$double.eps)

# Compute the generalized inverse of Q_pert

Q_inv <- ginv(as.matrix(Q_pert)) # Requires MASS package

#Compute the geometric mean of the variances, which are on the diagonal of Q.inv

scaling_factor = exp(mean(log(diag(Q_inv))))

## WRITE STAN MODEL (FOR TETRACYCLINE RESISTANT MRSA MODEL)

stan_data <- list(

N = nrow(east_cz_data), # Number of observations

J = length(unique(east_cz_data$CZ20)), # Number of czs

T = length(unique(east_cz_data$Year)), # Number of years

N_edges = nbs$N_edges, # Number of edges

node1 = nbs$node1, # Node 1 indices for edges

node2 = nbs$node2, # Node 2 indices for edges

cz = east_cz_data$cz_index, # cz indices

year = as.integer(as.factor(east_cz_data$Year)), # Year indices

tetra_count = east_cz_data$cz_tetra_count, # Response variable

log_offset = log(east_cz_data$expected_count), # Log offset term for population size (expected count)

scaling_factor = scaling_factor

)

# Hierarchical Bayesian Poisson model with BYM2

stan_model_code <- "

data {

int<lower=0> N; // Number of observations

int<lower=1> J; // Number of czs

int<lower=1> T; // Number of years

int<lower=0> N_edges; // Number of edges in the adjacency graph

int<lower=1, upper=J> node1[N_edges]; // Node 1 indices for edges

int<lower=1, upper=J> node2[N_edges]; // Node 2 indices for edges

int<lower=1, upper=J> cz[N]; // cz indices

int<lower=1, upper=T> year[N]; // Year indices

int<lower=0> tetra_count[N]; // Response variable

real log_offset[N]; // Offset term

real<lower=0> scaling_factor; // scales the variance of the spatial effects

}

parameters {

real alpha; // Intercept

real<lower=0, upper=1> rho; // Proportion of unstructured vs. spatially structured variance

real<lower=0> sigma; // overall standard deviation

vector[J] phi; // spatial effects

vector[J] theta; // Unstructured heterogeneous effects

vector[T] beta_year; // year effects

matrix[J, T] beta_interaction; // Interaction effects

}

transformed parameters {

vector[J] convolved_re; // BYM2 components

convolved_re = sigma * (sqrt(1 - rho) * theta + sqrt(rho / scaling_factor) * phi);

}

model {

// Priors

alpha ~ normal(0, 10); // Weakly informative prior for intercept

beta_year ~ normal(0, 10); // Weakly informative prior for year effects

to_vector(beta_interaction) ~ normal(0, 10); // Weakly informative prior for interaction effects

theta ~ normal(0, 1); // iid effect: neighborhood graph is fully connected

sigma ~ normal(0, 1); // normal prior for overall SD for combined BYM2 terms

rho ~ beta(0.5, 0.5); // Weakly informative prior favoring moderate spatial dependence

target += -0.5 * dot_self(phi[node1] - phi[node2]); // ICAR prior for phi

sum(phi) ~ normal(0, 0.001 * N); // soft sum-to-zero constraint on phi

// Likelihood (vectorized poisson model)

for (n in 1:N) {

tetra_count[n] ~ poisson_log(

alpha + convolved_re[cz[n]] + beta_year[year[n]] + beta_interaction[cz[n], year[n]] +

log_offset[n]

);

}

}

generated quantities {

vector[N] eta; // Store linear predictor

for (n in 1:N) {

eta[n] = alpha + convolved_re[cz[n]] + beta_year[year[n]] + beta_interaction[cz[n], year[n]];

}

}

"

## RUN STAN MODEL

#Save the Stan model to a file

stan_file <- "cz_year_rstan_bym2_tetra_model.stan"

writeLines(stan_model_code, con = stan_file)

# Compile the Stan model using rstan

rstan_options(auto_write = TRUE) #enables automatic writing of compiled Stan models (useful when repeatedly running model)

options(mc.cores = parallel::detectCores()) #sets number of cores for parallel computing, allows multiple MCs to run increasing efficiency

fit <- stan(

file = stan_file,

data = stan_data,

seed = 123,

chains = 3,

iter = 50000, #warmup and sampling

warmup = 25000,

cores= 3, #use parallel chains

control = list(max_treedepth = 15), #Increase tree depth

thin = 5,

init = 0.1 # Initialize all parameters around 0.1 (strictly positive)

)

saveRDS(fit, file = " cz_year_rstan_bym2_tetra_model.rds")

## MODEL DIAGNOSTICS

fit_summary<- summary(fit)#look at basic convergence diagnostics

#Check for convergence using Rhat

any(fit_summary$summary[, "Rhat"] > 1.01)

#Check for independent sampling using Effective Sample size (ESS)

any(fit_summary$summary[, "n_eff"] < 500)

**References**

1. Tolbert CM. Labor market areas for the United States: US Department of Agriculture, Economic Research Service, Agriculture and …; 1987.

2. Fowler CS. New Commuting Zone delineation for the US based on 2020 data. Scientific Data. 2024;11(1):975.

3. Tolbert CM, Sizer M. US commuting zones and labor market areas: A 1990 update. 1996.

4. Kaufman L, Rousseeuw PJ. Finding groups in data: an introduction to cluster analysis: John Wiley & Sons; 2009.

5. Li G, Haining R, Richardson S, Best N. Space–time variability in burglary risk: a Bayesian spatio-temporal modelling approach. Spatial Statistics. 2014;9:180-91.

6. Bernardinelli L, Clayton D, Pascutto C, Montomoli C, Ghislandi M, Songini M. Bayesian analysis of space—time variation in disease risk. Statistics in medicine. 1995;14(21‐22):2433-43.

7. Besag J, York J, Mollié A. Bayesian image restoration, with two applications in spatial statistics. Annals of the institute of statistical mathematics. 1991;43:1-20.

8. Simpson D, Rue H, Riebler A, Martins TG, Sørbye SH. Penalising model component complexity: A principled, practical approach to constructing priors. 2017.

9. Riebler A, Sørbye SH, Simpson D, Rue H. An intuitive Bayesian spatial model for disease mapping that accounts for scaling. Statistical methods in medical research. 2016;25(4):1145-65.

10. Morris M, Wheeler-Martin K, Simpson D, Mooney SJ, Gelman A, DiMaggio C. Bayesian hierarchical spatial models: Implementing the Besag York Mollié model in stan. Spatial and spatio-temporal epidemiology. 2019;31:100301.

11. Bivand RS, Wong DW. Comparing implementations of global and local indicators of spatial association. Test. 2018;27(3):716-48.

12. Stan Development T. RStan: the R interface to Stan. R package version 217 3. 2018.

13. Hoffman MD, Gelman A. The No-U-Turn sampler: adaptively setting path lengths in Hamiltonian Monte Carlo. J Mach Learn Res. 2014;15(1):1593-623.

14. Richardson S, Thomson A, Best N, Elliott P. Interpreting posterior relative risk estimates in disease-mapping studies. Environmental health perspectives. 2004;112(9):1016-25.
