# Supplementary material for: Spatiotemporal trends in tetracycline- and trimethoprim–sulfamethoxazole-resistant S. aureus among veteran outpatients in the eastern United States
Source: Epidemiol Infect. 2026 Feb 23;154:e31. doi: 10.1017/S0950268826101216 (PMC12976086; doi:10.1017/S0950268826101216)
Supplement: Boyle et al. supplementary material [file S0950268826101216sup001.zip › E&I_final_supplemental_tables.docx]

| Supplementary Table S1. Patient Exclusion Criteria | |
| --- | --- |
| Original raw VHA data | 558,737 |
| Excluded if <18 years of age | 3 |
| Excluded if geocoded residential address coordinates are not available | 1,027 |
| Excluded if geocoded residential address is not within the 48 contiguous states, DC, or has no valid State information | 5,277 |
| Excluded if missing antibiotic resistance results for all antibiotic classes | 490 |
| Excluded if missing MRSA classification | 6,474 |
| Excluded if not the first culture result for a given patient within a 30 day-period | 75,842 |
| Total after exclusion criteria | 469,624 |

| Supplementary Table S2. Alternative two-step classification of Commuting Zones based on their posterior probabilities of absolute relative risk and spatiotemporal trend. | | | | | | | | |
| --- | --- | --- | --- | --- | --- | --- | --- | --- |
|  | **Tetracycline Resistant MRSA** | | | | **TMP-SMX Resistant MRSA** | | | |
|  | High Risk | Moderate Risk | Low Risk | Total | High Risk | Moderate Risk | Low Risk | Total |
| Increasing | 0 | 39 | 14 | 53 | 0 | 24 | 17 | 41 |
| Stationary | 9 | 122 | 96 | 227 | 13 | 92 | 140 | 245 |
| Decreasing | 0 | 29 | 22 | 51 | 0 | 24 | 21 | 45 |
| Total | 9 | 190 | 132 | 331 | 13 | 140 | 178 | 331 |
| Alternative Classification Procedure based on posterior probabilities (PP): High risk= $\boldsymbol{PP>0.8}$, moderate risk= $\boldsymbol{0.2<}\boldsymbol{PP<0.8}$, and low risk= $\boldsymbol{PP<0.2}$. Increasing trend= $\boldsymbol{PP}\boldsymbol{>0.6}$, stationary trend= $\boldsymbol{0.4<PP}\boldsymbol{<0.6}$, and decreasing trend= $\boldsymbol{PP}\boldsymbol{<0.4}$. | | | | | | | | |

| **Supplementary Table S3:** Posterior differences in temporal effects between models with and without a space–time interaction. | | | | | | | | |
| --- | --- | --- | --- | --- | --- | --- | --- | --- |
|  | **Tetracycline Resistant MRSA** | | | | **TMP-SMX Resistant MRSA** | | | |
| **Year Index** | **Mean** | **Median** | **95% CI** | **Prob Diff >0** | **Mean** | **Median** | **95% CI** | **Prob Diff >0** |
| 1 | 0.83 | 0.82 | -10.50, 12.49 | 0.56 | 1.14 | 1.18 | -10.27, 12.37 | 0.58 |
| 2 | 0.15 | 0.15 | -11.13, 11.77 | 0.51 | 0.94 | 0.94 | -10.40, 12.19 | 0.56 |
| 3 | 0.93 | 0.92 | -10.43, 12.57 | 0.56 | 0.95 | 0.96 | -10.40, 12.25 | 0.57 |
| 4 | 0.79 | 0.80 | -10.54, 12.56 | 0.55 | 0.48 | 0.51 | -11.00, 11.63 | 0.53 |
| 5 | 1.22 | 1.23 | -10.07, 12.87 | 0.58 | 1.07 | 1.07 | -10.28, 12.28 | 0.58 |

| **Supplementary Table S4:** Posterior differences in temporal effects between models with the original priors and RW1 priors | | | | | | | | |
| --- | --- | --- | --- | --- | --- | --- | --- | --- |
|  | **Tetracycline Resistant MRSA** | | | | **TMP-SMX Resistant MRSA** | | | |
| **Year Index** | **Mean** | **Medium** | **95% CI** | **Prob Diff >0** | **Mean** | **Medium** | **95% CI** | **Prob Diff >0** |
| 1 | 1.13 | 1.14 | -7.19, 9.57 | 0.60 | 1.53 | 1.53 | -6.99, 9.92 | 0.64 |
| 2 | 0.44 | 0.43 | -7.91, 8.92 | 0.54 | 1.31 | 1.30 | -7.18, 9.79 | 0.62 |
| 3 | 1.22 | 1.21 | -7.23, 9.68 | 0.61 | 1.33 | 1.29 | -7.16, 9.87 | 0.62 |
| 4 | 1.07 | 1.03 | -7.20, 9.52 | 0.60 | 0.87 | 0.86 | -7.63, 9.32 | 0.58 |
| 5 | 1.49 | 1.47 | -6.82, 9.92 | 0.63 | 1.45 | 1.44 | -6.99, 9.88 | 0.63 |

| Supplementary Table S5: Two-step classification of Commuting Zones using RW1 prior specifications (original results are in parentheses). | | | | | | | | |
| --- | --- | --- | --- | --- | --- | --- | --- | --- |
|  | **Tetracycline Resistant MRSA** | | | | **TMP-SMX Resistant MRSA** | | | |
|  | **High Risk** | **Moderate Risk** | **Low Risk** | **Total** | **High Risk** | **Moderate Risk** | **Low Risk** | **Total** |
| Increasing | 0 (0) | 0 (1) | 0 (0) | 0 (1) | 0 (0) | 0 (0) | 0 (0) | 0 (0) |
| Stationary | 36 (9) | 147 (189) | 148 (132) | 331 (330) | 38 (13) | 96 (140) | 197 (178) | 331 (331) |
| Decreasing | 0 (0) | 0 (0) | 0 (0) | 0 (0) | 0 (0) | 0 (0) | 0 (0) | 0 (0) |
| Total | 36 (9) | 147 (190) | 148 (132) | 331 (331) | 38 (13) | 96 (140) | 197 (177) | 331 (331) |
